# Forelimb Kinematics of Rats Using XROMM, with Implications for Small Eutherians and Their Fossil Relatives

**DOI:** 10.1371/journal.pone.0149377

**Published:** 2016-03-02

**Authors:** Matthew F. Bonnan, Jason Shulman, Radha Varadharajan, Corey Gilbert, Mary Wilkes, Angela Horner, Elizabeth Brainerd

**Affiliations:** 1 Biology Program, Stockton University, Galloway, New Jersey, 08205, United States of America; 2 Physics Program, Stockton University, Galloway, New Jersey, 08205, United States of America; 3 Department of Biology, California State University, San Bernardino, California, 92407, United States of America; 4 Department of Ecology and Evolutionary Biology, Brown University, Providence, Rhode Island, 02912, United States of America; University of Zurich, SWITZERLAND

## Abstract

The earliest eutherian mammals were small-bodied locomotor generalists with a forelimb morphology that strongly resembles that of extant rats. Understanding the kinematics of the humerus, radius, and ulna of extant rats can inform and constrain hypotheses concerning typical posture and mobility in early eutherian forelimbs. The locomotion of *Rattus norvegicus* has been extensively studied, but the three-dimensional kinematics of the bones themselves remains under-explored. Here, for the first time, we use markerless XROMM (Scientific Rotoscoping) to explore the three-dimensional long bone movements in *Rattus norvegicus* during a normal, symmetrical gait (walking). Our data show a basic kinematic profile that agrees with previous studies on rats and other small therians: rats maintain a crouched forelimb posture throughout the step cycle, and the ulna is confined to flexion/extension in a parasagittal plane. However, our three-dimensional data illuminate long-axis rotation (LAR) movements for both the humerus and the radius for the first time. Medial LAR of the humerus throughout stance maintains an adducted elbow with a caudally-facing olecranon process, which in turn maintains a cranially-directed manus orientation (pronation). The radius also shows significant LAR correlated with manus pronation and supination. Moreover, we report that elbow flexion and manus orientation are correlated in *R*. *norvegicus*: as the elbow angle becomes more acute, manus supination increases. Our data also suggest that manus pronation and orientation in *R*. *norvegicus* rely on a divided system of labor between the ulna and radius. Given that the radius follows the flexion and extension trajectory of the ulna, it must rotate at the elbow (on the capitulum) so that during the stance phase its distal end lies medial to ulna, ensuring that the manus remains pronated while the forelimb is supporting the body. We suggest that forelimb posture and kinematics in *Juramaia*, *Eomaia*, and other basal eutherians were grossly similar to those of rats, and that humerus and radius LAR may have always played a significant role in forelimb and manus posture in small eutherian mammals.

## Introduction

The evolutionary ecology of Mesozoic mammals is often inferred from skeletal traits associated with locomotion. In the past 25 years, several post-cranial fossils from mammaliforms, theriiomorphs, and basal therian mammals have yielded diverse appendicular morphologies inferred as reflecting habits ranging from arboreal to fossorial [1–11]. Compared with the (often) conservative morphology of the hindlimb, the morphologies of the forelimb skeleton from scapula to manus are more diverse [12,13]. Following a long tradition of form-function studies, the skeletal morphology of Mesozoic mammal forelimbs has been used to support various ecomorphological hypotheses of locomotion [12,13].

In this context, small-bodied therian mammals have often been utilized to decipher the locomotor habits of their ancestors given the similarities in their appendicular morphologies. Classic studies by Jenkins and colleagues [14–20] utilized a combination of radiographic stills, cineradiography, and the comparative method to deduce the probable locomotor habits of early therians and theriiomorphs. This body of research suggested that many early therians were scansorial to arboreal in their habits. More recently, Fischer and colleagues [21,22] demonstrated that for small therian mammals, forelimb posture and kinematics followed a conservative pattern: the forelimb remains crouched but parasagittal, forming a three segment zigzag (scapula, humerus, forearm + manus), and scapular movement contributes the most to step length. Fischer and colleagues [21,22] suggested that given the conserved nature of this posture across a large phylogenetic cross-section of small therians, Mesozoic therians probably utilized similar gaits. Moreover, it is likely that a crouched but parasagittal forelimb posture was primitive for basal therians regardless of their locomotor habits [21,22].

Although the broad aspects of small therian mammal forelimb kinematics are well delineated, other questions remain. Manus pronation is often assumed to play a significant role in parasagittal locomotion by ensuring that manus flexion and extension occurs in line with the direction of travel [23,24]. Manus pronation in a variety of therian mammals is often accomplished via long-axis rotation of the radius about the ulna [25]. In fact, radial head shape and shaft curvature are often correlated with the ability to pronate the manus [24]. Understanding the extent and degree to which long-axis rotation of the radius could occur in early eutherians, for example, would further illuminate and constrain their potential locomotor envelopes. Moreover, the well-preserved forelimbs of the two earliest known eutherians, *Juramaia* and *Eomaia*, suggest these mammals were scansorial locomotor generalists, with the latter animal inferred to be more arboreal in its habits [4,7]. In many arboreal species of mammals, and in arboreal reptiles such as chameleons, prehensile appendages are critical aspects of navigating narrow perches [26]. Given the continuing uncertainties surrounding the primitive locomotor habits of early therians, it would be informative to understand the three-dimensional kinematics of the humerus, radius, and ulna *in vivo* during locomotion in small eutherian mammals.

Among several model animals, the locomotion and posture of *Rattus norvegicus* is perhaps the most thoroughly investigated of all small-bodied scansorial mammals [27]. In particular, the kinematics of locomotion and reaching behaviors in this species are extensively documented [28–36]. Collectively, these studies and others have shown that the greatest forelimb movements occur proximally, but that rats are capable of pronating their manus. Without three-dimensional kinematic bone data, however, it has been difficult to determine if manus pronation in rats is due to movements of the radius and ulna within the forearm, or whether such movements are simply the result of long-axis rotation (LAR) and abduction in more proximal elements [28–30]. In fact, understanding the impact of LAR on posture and locomotion in tetrapods has remained difficult at best, and the LAR for rat forelimbs is currently unknown. However, LAR must play a significant but poorly appreciated role in tetrapod locomotion. For example, recent three-dimensional kinematic studies of bird limbs show that many maneuvers cannot occur without significant LAR of the tibiotarsus [37,38].

X-ray Reconstruction of Moving Morphology (XROMM) is an ideal tool to explore three-dimensional bone movements *in vivo* from hi-speed biplanar radiographic videos [39,40]. Here, we explore the three-dimensional movements of the humerus, radius, and ulna *in vivo* in *Rattus norvegicus* during a normal, symmetrical gait (walking). Given the small size of the manus in rats, we were unable to directly track hand movements. However, long-axis rotation of the radius is correlated with manus pronation in eutherian mammals [24,25]. Therefore, movement of the radius serves as a proxy measure of manus pronation. Using the markerless XROMM approach called Scientific Rotoscoping (SR), we analyzed the three-dimensional kinematics of the humerus, radius, and ulna in three male Sprague-Dawley rats as they walked across a flat platform. Our data are then compared with previous studies on rat and small mammal forelimb kinematics, followed by a discussion of the paleobiological implications of our results.

## Materials and Methods

### Animals

All research methods and animal usage were approved by the Institutional Animal Care and Usage Committees (IACUC) at Stockton University (SU) and Brown University, and housing and feeding procedures strictly followed recognized IACUC guidelines for rats [41]. This study also followed ARRIVE guidelines [42]. Three young (10–12 weeks old) male Sprague-Dawley rats (Rat1, Rat2, and Rat3 hereafter), each weighing approximately 325–375 g, were selected for this research. A fourth, older (18 months) male Sprague-Dawley rat (Rat4 hereafter), approximately 400 g, was sacrificed and implanted with tantalum beads to test our digitizing accuracy (see below). All statistical analyses and graphs generated from our data were analyzed using MATLAB [43], SPSS [44], and the XMA software developed at Brown University and available on the XROMM website (www.xromm.org).

### Cineradiography, Bone Model Processing, and Scientific Rotoscoping

The goal of XROMM is to animate and reconstruct the three-dimensional movements of the skeleton. We followed the standard workflow for manual markerless XROMM (Scientific Rotoscoping; [40]): 1) capture of two calibrated, synchronized radiographic videos of animal movement; 2) building of separate polygonal mesh models of each bone from CT scans; 3) construction of a digital marionette [40] of the skeleton elements of interest; 4) reconstruction of the scene in animation software (Autodesk Maya [45]) using calibration data to position virtual cameras that project the frames of the radiographic video onto virtual screens; and 5) registration of the skeletal marionette via posing the model against the X-ray images on each frame of the video [40,46]. DICOM files of each rat and STL files are available through the XMA Portal (http://xmaportal.org/webportal/).

**Fig 1:**
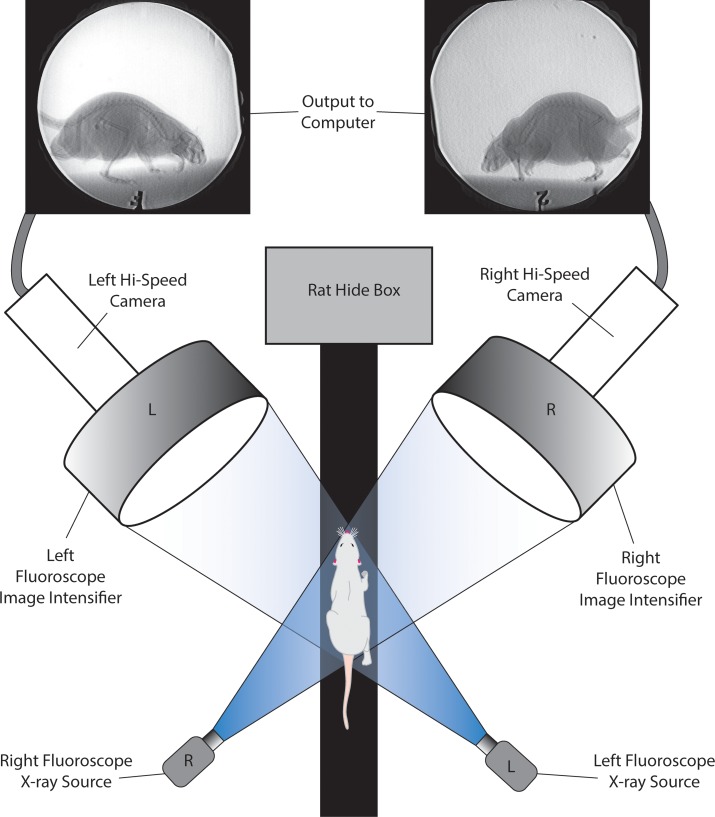
Our XROMM setup for capturing skeletal movements of walking rats. Rats were trained to walk down a narrow trackway towards a dark hide box placed on the other side of the two C-arm videofluoroscopes.

In the C-arm fluoroscope lab at Brown University, the three young male rats were trained to move across a 10 cm wide and 5 meter long flat platform covered with a roughened fabric to provide traction (see Fig 1). Rats naturally seek out dark shelter and were trained to walk the length of the platform to hide in a small, darkened box. Two mobile C-arm videofluoroscopes were positioned on either side of the platform near the hide box. In this way, we were more likely to capture natural movements of the forelimbs in action rather than movements related to braking or initializing locomotion. Given that our goal was to capture long-axis rotation of the radius (pronation), each C-arm videofluoroscope was positioned obliquely lateral on the left and right sides of the track. Each C-arm videofluoroscope component is a modified OEC 9400 Medical Systems model with a 30 cm image intensifier. During each trial, X-ray emission was continuous at 70 kV and 20mA in boost mode. Each videofluoroscope is connected to a Photron 1024 PCI high-speed camera which recorded videos at 250 frames per second at a 1/1000 shutter speed at high resolution (1024 X 1024). At the beginning and end of each set of trials, a calibration object was filmed through both videofluoroscopes. The object consists of 48 steel spheres (3 mm diameter) that function as registration points evenly distributed throughout an acrylic, three-tiered cube designed for previous XROMM trials (see [39]). A total of 26 trials of the three rats were captured; of these, the 10 best trials were selected for XROMM analysis.

After the trials were completed, the three male rats were euthanized and CT-scanned to capture their three-dimensional skeletal morphology. These scans were subsequently imported into the Slicer3D program (www.slicer.org; [47]) and STL mesh models of the left humerus, radius, ulna, skull, and sternum were exported. The programs OpenFlipper (www.openflipper.org; [48]) and MeshLab (meshlab.sourceforge.net; [49]) were utilized for cleanup and additional processing of the mesh files before import into the Autodesk Maya software environment (see below). Standard XROMM image processing procedures were followed to remove fluoroscope distortion from the captured videos, and registration of points between the two views of the calibration object were used to calculate the virtual camera angles in Autodesk Maya [39,40].

We chose to reconstruct forelimb movements using Scientific Rotoscoping (SR), which is a manual, markerless method of XROMM [40,46]. In SR, digital marionettes of the bones are matched (registered) to their X-ray images simultaneously in two views (one view for each videofluoroscope) for each frame of the animation (see below). The Autodesk Maya [45] software environment, in combination with XROMM X-ray Project Tools (www.xromm.org), was used to construct a digital marionette of the left forelimb from the CT-scan data of each rat: humerus, radius, and ulna.

The digital marionettes were constructed following SR protocols described by [40] and [46] and by utilizing XROMM Maya Tools (available from xrommwiki.org) in Autodesk Maya. Each bone was modeled separately from the CT data of each rat, in which the front half of the animal was scanned. This allowed our models to retain positional information relative to one another and the rat’s body. This was important because it allowed us to import bones into Autodesk MAYA in their natural, articulated positions. Three-dimensional movements of the scapula could not be reconstructed because this element is very thin and flat, and its X-ray profile in the videos was faint and often undiscernible. Whereas the scapula is a major contributor to forelimb movements, our inability to precisely orient this proximal bone would have compounded our error in positioning the remainder of the forelimb model. Therefore, given that the humerus could be matched confidently to its *in vivo* orientations from all biplanar cineradiographic videos, we selected this element as the proximal-most bone in our kinematic marionette. Moreover, we confirmed that accurately reconstructing humerus movements without reference to the scapula were possible via a gold standard accuracy test (see below). We plan to track the scapula in future studies using radio-opaque markers.

**Fig 2:**
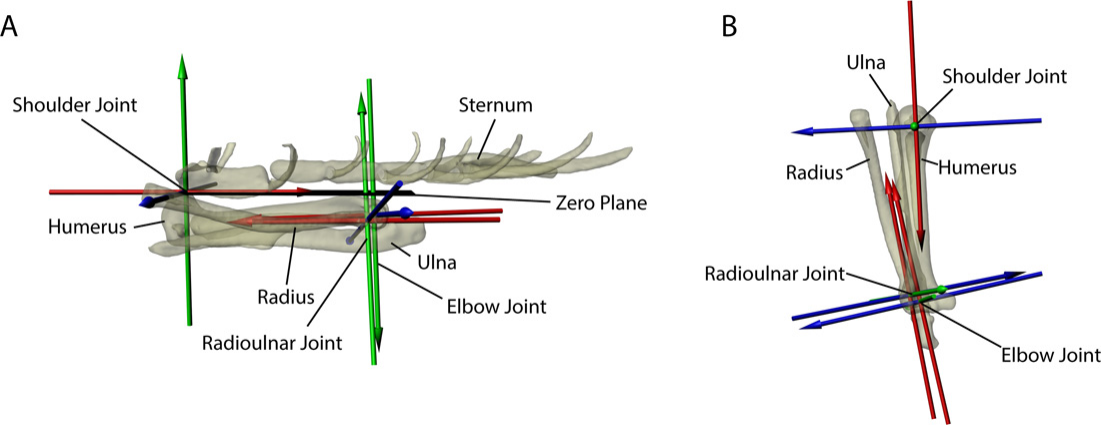
The reference frame (zero position) of the left forelimb rig in (A) left lateral and (B) dorsal views. The bones have been made semi-translucent to enhance visualization of the three Joint Coordinate Systems (JCS) that comprised the virtual shoulder, elbow, and radioulnar joints. All angles and translations are recorded in relation to the reference frame. Note that the reference frame is not intended to be a natural orientation: in fact, in this orientation the radius is unnaturally rotated so that it is entirely supinated relative to the ulna, a posture impossible for a rat. However, this reference posture provides a point against which the degree of pronation can be quantified. To account for body movements, the shoulder JCS has the sternal manubrium centered on the caudal humeral head. Each JCS was based on an Euler angle ZYX rotation order that followed the right-hand rule. In this way, movement of the Z-axis also moved the Y- and X-axes as well as the bone model; movement of Y-axis also moved the X-axis and the bone model; and movement of the X-axis only moved the bone model.

The forelimb rig linked the left humerus, radius, and ulna together in a kinematic chain (see Fig 2). Following Gatesy and colleagues [40], this allows the bones to retain their natural articulations and spacing, and facilitates registration of the models with their X-ray images. For each video sequence, the proximal-most element, the humerus, was first aligned and registered with each frame, followed by the ulna, and finally the radius. Without a body frame of reference, movements at the shoulder could not be tracked independently of body movement itself. In other words, how do we know that the movements we report for the shoulder are intrinsic or a combination of body and shoulder movements? Therefore, we constructed a second marionette rig consisting of the skull and sternum with which we compared the movements of the shoulder. The skull was clearly visible in each cineradiographic video and could be confidently registered for the frames of each video sequence. We linked the sternum, which was more translucent to X-rays, to the skull in our rig so we could ensure that by reconstructing head movements the midline of the chest could be approximated. This approach is similar to other protocols that bracket elements nearly translucent to X-rays within confidently-registered ones in previous XROMM studies [46].

Each left forelimb rig was placed into a predefined zero position (a reference frame) in which all joint translations and rotations are set to zero (see Fig 2). All translations and degrees of rotation reported here are derived in relation to this reference frame (see Fig 2). The zero position places the humerus in parallel beneath a horizontal plane, with the caudal-most aspect of the humeral head centered beneath the manubrium of the sternum. The ulna was positioned such that its olecranon process aligned with the center of the humeral shaft. The radius was rotated at its proximal end 180° from its most pronated orientation (as determined from the cineradiographic videos) relative to the ulna so that it was supinated, mimicking human anatomical position. This reference frame orientation is not anatomically feasible but rather was done to standardize the analysis and discussion of the reported movements.

**Fig 3:**
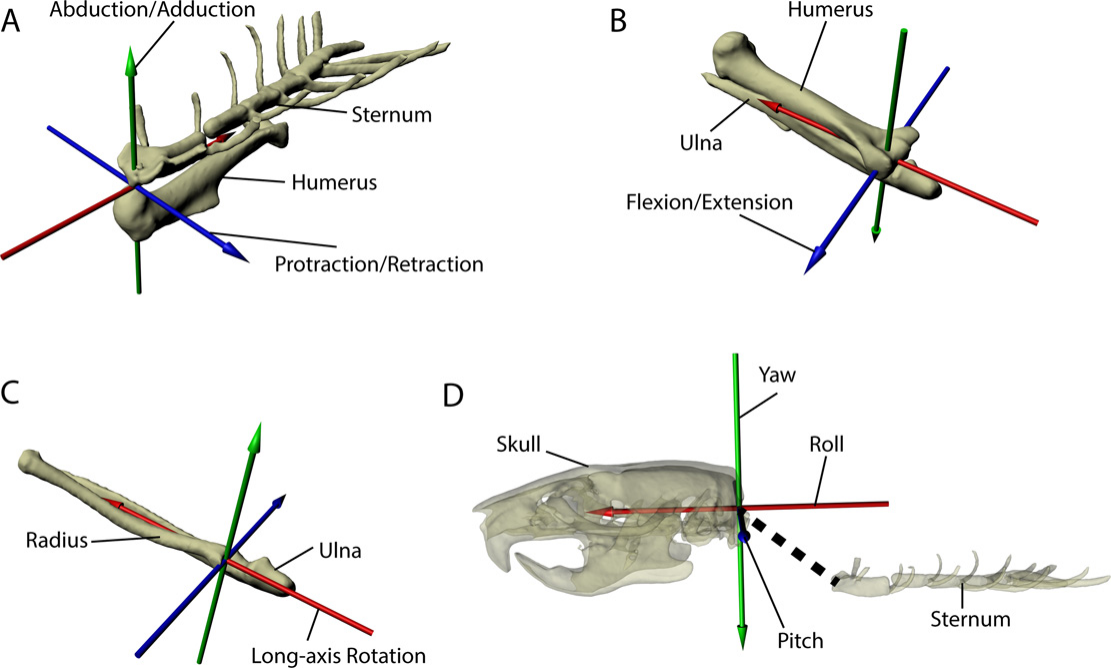
Joint coordinate systems illustrated. Each joint coordinate system (JCS) of the forelimb as well as the JCS of the skull and sternum utilized in the gold standard accuracy test are isolated and illustrated to show greater detail. Each JCS was based on an Euler angle ZYX rotation order that followed the right-hand rule. For all JCS, the Z-axis is blue, the Y-axis is green, and the X-axis is red. For all forelimb JCS, the Y-axis measures abduction/adduction and the X-axis measures long-axis rotation (LAR). At the shoulder JCS (A) shown in oblique left cranial view, the Z-axis measures protraction/retraction of the humerus relative to the sternum. At the elbow JCS (B) and the radioulnar JCS (C) (both shown in oblique caudodorsal view), the Z-axis measures flexion/extension of the ulna relative to the humerus or of the radius relative to ulna, respectively. To determine the body midline frame of reference, the skull and sternum were linked together as a separate rig (D). A skull-sternum JCS was created for the gold standard to test how accurately manual registration of the skull allowed us to determine the midline of the body using the sternum as a reference. The skull and sternum rig is shown in left lateral view and the bones are semi-translucent to facilitate visualization of the skull-sternum JCS. For the skull-sternum JCS (D), the pitch (Z-axis), yaw (Y-axis), and roll (X-axis) of the sternum was measured relative to the skull.

For the forelimb marionette rig, joint coordinate systems (JCS), or virtual joints, with six degrees of freedom (6 DOF) were created in three locations (shoulder, elbow, radioulnar joint) on the reference frame to track the movements of the bones relative to one another (Figs 2 and 3). Each JCS was based on an Euler angle ZYX rotation order that followed the right-hand rule. In this way, movement of the Z-axis also moved the Y- and X-axes as well as the bone model; movement of Y-axis also moved the X-axis and the bone model; and movement of the X-axis only moved the bone model. We purposefully created each JCS with 6 DOF so as not to bias our interpretation of joint movements *a priori*. The registration of the models with their X-ray videos ultimately decided the *in vivo* range of movement available at each JCS.

The shoulder JCS was centered directly between the manubrium of the sternum and the caudal-most aspect of the humeral head, and measured the movement of the humerus relative to the sternum: protraction/retraction (Z-axis), abduction/adduction (Y-axis), and long-axis rotation (LAR) (X-axis) (Figs 2 and 3). The X-axis of the shoulder JCS points caudally through the center of the olecranon fossa (Figs 2 and 3). The elbow JCS was situated between the epicondyles of the humerus proximally and the semi-lunar notch of the ulna distally, and measured the movement of the ulna relative to the humerus: flexion/extension (Z-axis), abduction/adduction (Y-axis), and LAR (X-axis) (Figs 2 and 3). Finally, the radioulnar JCS was centered on the proximal end of the radius, and measured the movement of the radius relative to the ulna: flexion/extension (Z-axis), abduction/adduction (Y-axis), and LAR (X-axis) (Figs 2 and 3).

For each JCS, the movements we describe follow standard veterinary anatomical conventions for quadrupedal mammals [50]. At the shoulder, we use the term protraction to indicate cranial rotation of the humerus, and retraction to indicate caudal rotation. Movements ventral and lateral to the zero position at this joint are reported in negative degrees to indicate that the humerus is moving below the horizontal or away from the body wall. For example, as the shoulder protracts the humerus is depressed below the horizontal, resulting in negative rotational degrees, whereas maximal retraction yields positive rotational degrees when the humerus is elevated above the horizontal; abduction also results in negative rotational degrees. For the long-axis rotation (LAR) of the humerus about the shoulder, increasingly negative values indicate medial rotation whereas increasingly positive values show lateral rotation. For the elbow, values less than 90° indicate a flexed elbow, whereas those 90° or greater indicate the elbow is extended. LAR of the radius relative to the ulna is defined here as radius pronation: at the radioulnar JCS, pronation increases, and supination decreases, as its value approaches 180°. Hereafter, our use of the terms pronation and supination refer strictly to the long-axis rotation of the radius about the ulna unless otherwise indicated.

### Measuring Accuracy and Precision

Given that the SR approach to XROMM involves manual registration of the bone models to the radiographic videos, we wanted to ensure that our reconstructions and the kinematic data reported here were both accurate and precise. Following previous XROMM studies [39,51], we devised a gold standard against which to compare our SR reconstructions for accuracy. The bones of a cadaver rat (Rat4) were implanted with 0.8 mm tantalum beads (markers hereafter) that could be tracked with XMA Lab software (www.xromm.org/xmalab). Multiple markers were implanted in the skull, sternum, scapula, humerus, ulna, and radius of Rat4 so that the full six degrees of freedom (6DOF) of each JCS could be tracked. The specimen was then secured to the same platform used in the trials and rigged with a string that could be pulled to simulate protraction/retraction of the forelimb including flexion/extension at the elbow. Given that the radius was implanted with markers independently of the ulna, it was also possible to track how the former bone moved in relation to the latter.

Using XMA Lab software, bone movements were automatically tracked over 80 frames which encompassed a sequence of the forelimb moving from a retracted to a protracted orientation, flexion to extension of the elbow, and long-axis rotation of the humerus and radius. This analysis does not depend on the specific locations of the beads. Instead, the markers were placed non-collinearly and used to “drive” the animations of the bones via rigid-body modeling. This generated known orientations of the sternum JCS, the shoulder JCS, the elbow JCS, and the radioulnar JCS. These known JCS orientations were our gold standard. In a new animation of these same video frames, the forelimb rig of Rat4 was registered to the video sequences manually. As per previous XROMM studies [39,51], working in Autodesk Maya makes it possible to use exactly the same reference pose and JCS for both the marker-driven gold standard and the markerless analysis. To ensure we were not matching our forelimb rig to the radiopaque markers visible in the original video, we created duplicate videos in which we digitally erased these markers in Photoshop for each of the 80 frames as per Baier and colleagues [51]. After we successfully registered our forelimb rig manually, we exported the translational and rotational ZYX coordinates of the same JCS for comparison with our tracked marker data. Given that we used the sternum as our body midline reference, the skull-sternum JCS was specifically created for the gold standard as a test of how accurately manual registration of the skull could orient the sternum (Fig 3). The skull-sternum JCS was centered on the foramen magnum and measured the movement of the sternum relative to the skull: pitch (Z-axis), yaw (Y-axis), and LAR (X-axis).

The mean residuals of the translational and rotational orientations at the joints between the gold standard (the known orientations) and SR manual registration give us our measure of accuracy. Translational accuracy for the skull/sternum JCS was 0.6–1.3 mm, with rotational accuracies of 0.4–1.8° (S1 Table). Translational accuracy at each forelimb JCS was submillimeter for all measures (S1 Table), and rotational accuracy at each forelimb JCS was within 1.4–3.6° (S1 Table), results similar to those of other SR XROMM studies [51]. We also tested our manual registration precision using a repeated measures approach with markerless data similar to that proposed by Gatesy and colleagues [40]. In this case, we chose a single frame from a sequence for each rat and manually registered our models to that frame 10 times. For each registration, the model was returned to its zero position and re-registered to the frame from scratch. The data from each re-registered frame for each rat was subsequently exported and compared. We tested the most proximal and distal joints in our rigs: the shoulder and the radiolunlar JCS. Precision of the shoulder is crucial because both its translations and rotations substantially affect the orientation of the radius and ulna elements distal to the humerus. Precision of the radiolunlar JCS was also critical to demonstrate whether significant long-axis rotation of the radius occurs relative to ulna, or at least whether it could reasonably be detected. As S2 Table shows, at the shoulder JCS, we were precise to submillimeter levels, and to within 2 degrees for rotational orientation. For the radioulnar JCS, our precision fell within 1 degree of rotation. Therefore, we can be confident that our manual registration method (SR) of reconstructing forelimb movements is both accurate and precise to within less than a millimeter of translation and 1.4–3.6° of rotation at each forelimb JCS.

### Calculating the Limb Cycle

Given that we were quantifying forelimb bone movements *in vivo* during normal walking in Sprague-Dawley rats, we did not utilize a treadmill. Although the kinematics of walking on a static platform and walking on a treadmill can sometimes yield similar results [21], the three-dimensional kinematics of the forelimb bones in *Rattus norvegicus*, especially their LAR, remain poorly understood. Therefore, because we had no *a priori* means of knowing how treadmill walking would affect our data, we chose to examine walking on a static platform to eliminate any additional variables. This allows us to establish a typical, three-dimensional kinematic pattern which can be compared to future treadmill studies.

However, the advantage of utilizing a treadmill is that it acts to keep a conditioned animal in front of cameras or fluoroscopes so that several limb cycles can be tracked. Since there is no reliable or practical way to physically move the videofluoroscopes in relation to the rats, our videos consist of static views through which the rat subjects pass from left to right (or right to left) across the screen. Due to the small field of view captured by the videofluoroscopes, it was usually not possible to cleanly capture a full step cycle start to finish, and our videos often start and end in the middle of a step cycle. Moreover, because the rats were not walking on a treadmill at a standardized speed, direct comparisons of their limb cycles was not possible.

Therefore, to enable us to compare the data from our three rats over the ten best trials, we standardized the step cycle. We compared data across individuals and trials by calculating events relative to the portion of the stride in stance and swing. We chose to track the distal end of the ulna because, unlike the radius, this bone only moves significantly in flexion-extension (see Results) and serves as a proxy for the location of the wrist. This method allowed us to determine periods when the distal end of the ulna was static, *i*.*e*. the stance phases. We found that the stance and swing phases represented approximately 64% and 36% of the step cycle, respectively. This allowed us to calculate the portion of the step cycle represented by each frame of the video. A typical stance phase begins with touchdown of the manus and ends just prior to toe-off with the metacarpus held vertically and the most distal phalanges maximally extended. This is followed by the swing phase which begins at toe-off and ends just prior to manus touchdown. All joint excursion data for our 10 trials is available in the Supporting Information (S1 Data).

Given the large number of overlapping data points and the inherent variation of the step cycle across the rats and trials, we examined both individual rats and a combined, averaged step cycle. Each graph charts the percentage of the step cycle on the horizontal (X) axis, and the degrees of joint rotation on the vertical (Y) axis. For the averaged step cycle, on the X-axis, we divided our step cycle data into 20 equal duration bins, each representing 5% of the step cycle.

## Results

**Fig 4:**
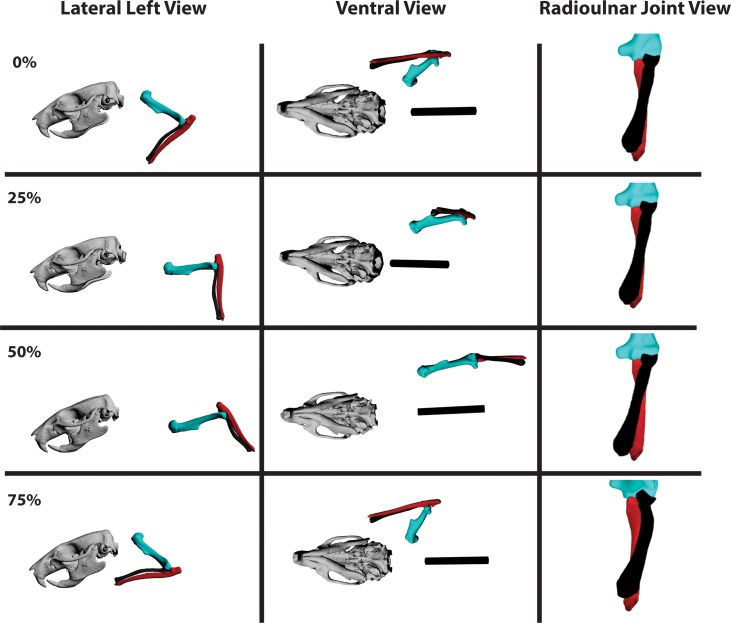
Lateral, ventral, and radioulnar joint views of the humerus (sea green), radius (black), and ulna (red) in a typical step cycle in *Rattus norvegicus*. Long-axis rotation (LAR) of the radius about the ulna (radius pronation) is shown in cranial view from the perspective of the ulna (the ulna appears to be stationary in the radioulnar joint view relative to the humerus and radius). Note radius (black) LAR relative to the ulna (red). Percentages = portion of the step cycle. Black bar in ventral view = body midline based on sternum.

**Fig 5:**
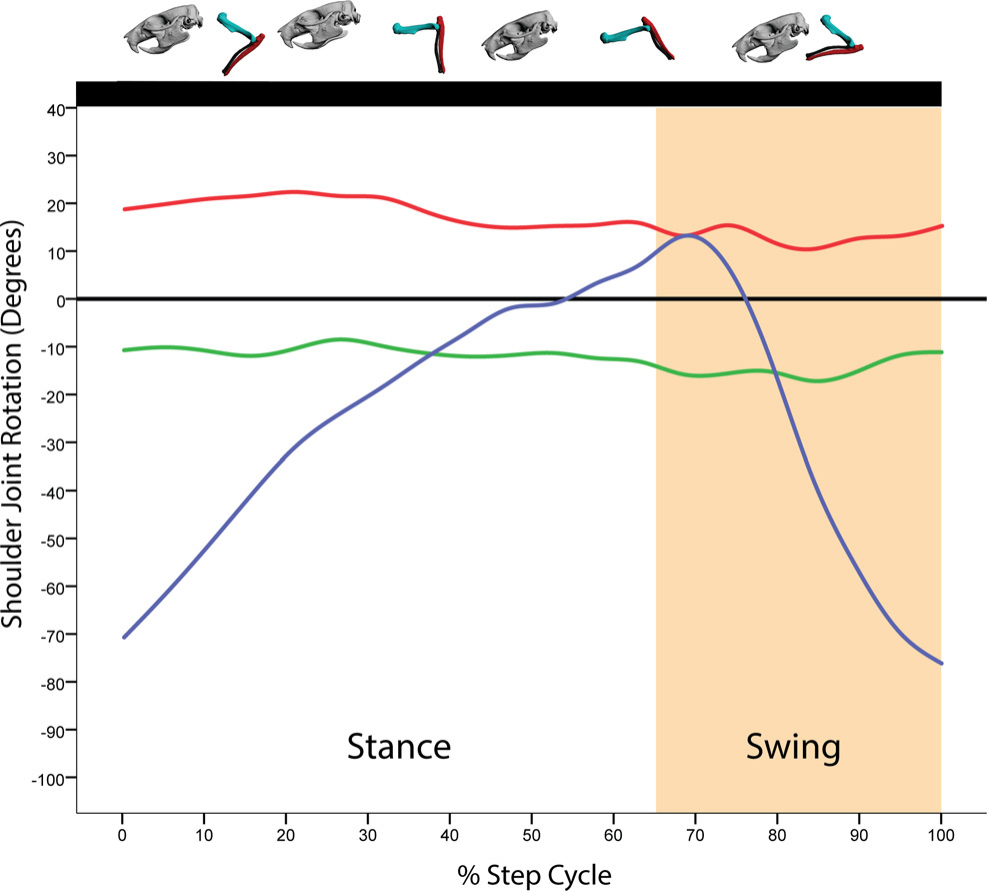
Average motion in degrees at the shoulder joint coordinate system (JCS) for a *R*. *norvegicus* step cycle. Above the graph is a representation of the forelimb posture relative to the step cycle. Here, all ten trials from all three rats were binned for every 5% of the step cycle. Blue = Z-axis (protraction/retraction); Green = Y-axis (abduction/adduction); X-axis (long-axis rotation).

**Fig 6:**
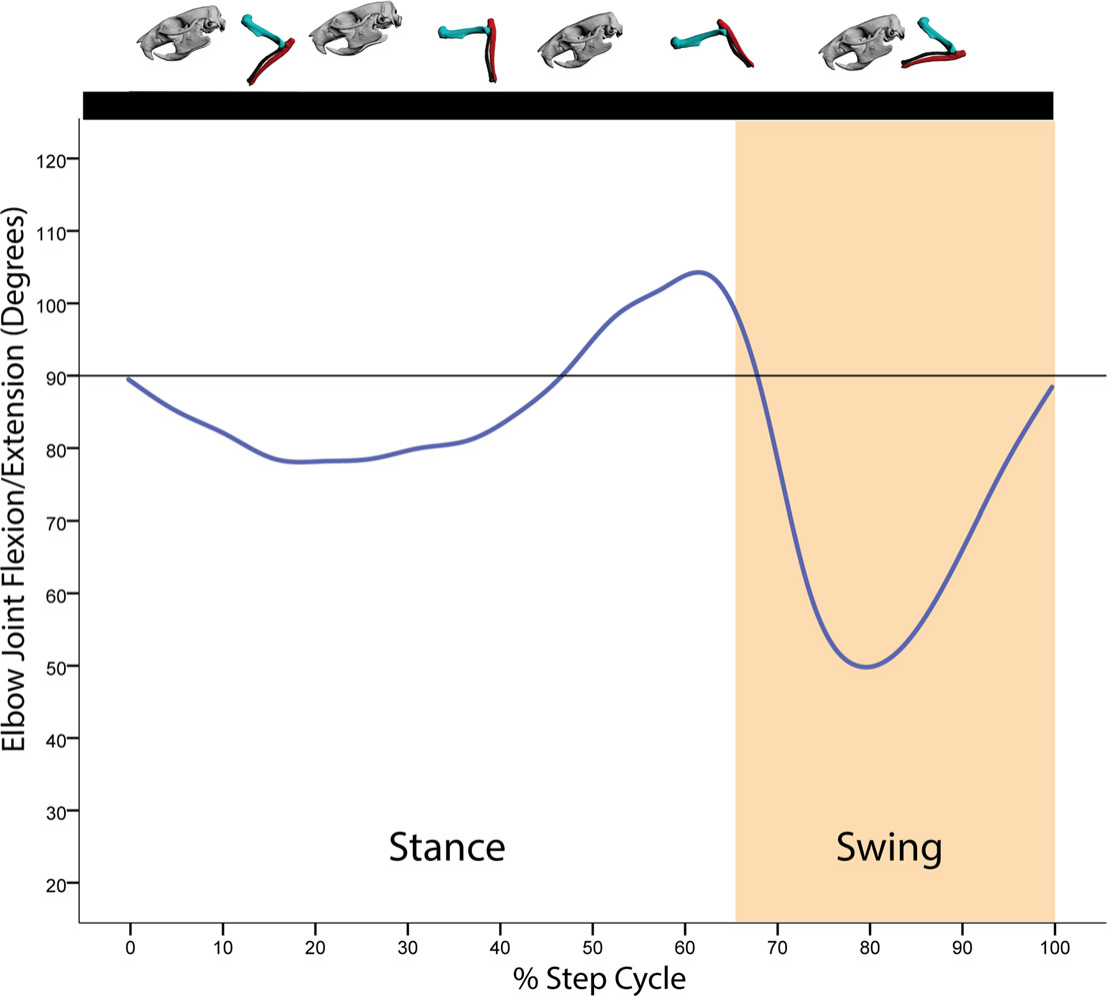
Average motion in degrees at the elbow joint coordinate system (JCS) for a *R*. *norvegicus* step cycle. Above the graph is a representation of the forelimb posture relative to the step cycle. Here, all ten trials from all three rats were binned for every 5% of the step cycle. Given the tiny rotational movements at the Y- and X-axes for the elbow JCS, only the Z-axis rotations are shown. Blue = Z-axis (flexion/extension).

**Fig 7:**
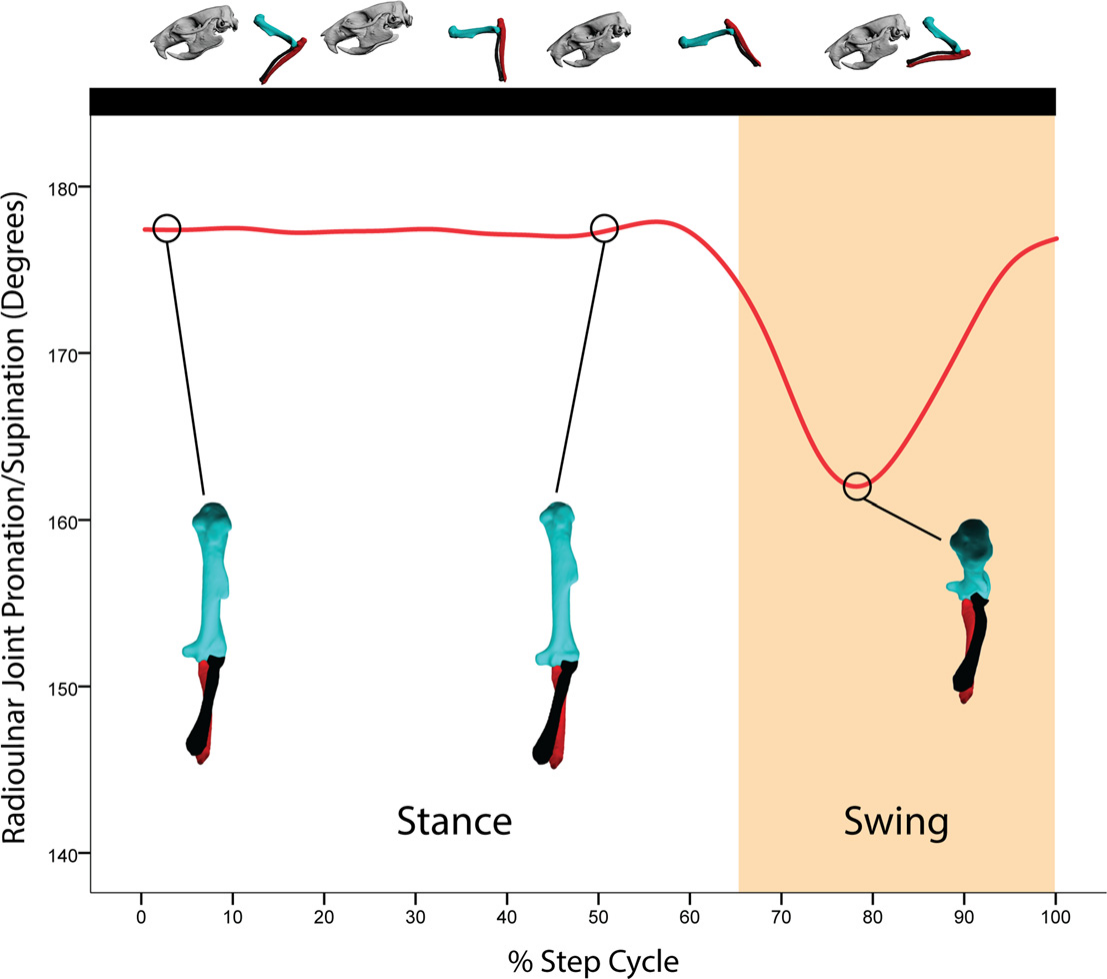
Average motion in degrees at the radioulnar joint coordinate system (JCS) for a *R*. *norvegicus* step cycle. Above the graph is a representation of the forelimb posture relative to the step cycle. Here, all ten trials from all three rats were binned for every 5% of the step cycle. Radius long axis rotation (LAR, pronation) is shown in cranial view from the perspective of the ulna (the ulna appears to be stationary in these figures compared to the humerus and radius). Note the close synchrony between elbow flexion (Fig 7) and radius LAR. Given the small rotational movements at the Z- and Y-axes for the radioulnar JCS, only the X-axis rotations are shown. Red = X-axis (radius LAR relative to the ulna).

The basic 2-D kinematic profile of the forelimb joints in our walking rats agrees with previous analyses [20,21]. As observed, noted, and recorded for rats and other small therian mammals [20,21,52], the forelimb retained a crouched but parasagittal posture throughout the step cycle. For the following descriptions refer to Fig 4 for left lateral, ventral, and cranial elbow views of a typical step cycle. Table 1 shows joint angle excursion data recorded for each joint in degrees as well as minimum, maximum, mean, and median values. Figs 5–7 show the averaged step cycle based on the 10 trials. Additional figures showing the averaged step cycle and its standard deviation as well as a typical step cycle for a single rat are available in the Supporting Information (S1–S6 Figs). The combined radiographic videos and animated polygonal bone models of all trials through both video fluoroscopes are available for viewing in the Supporting Information (S1 Video). A single reconstructed and looped step cycle from the Rat2 Trial 1 data can also be viewed (S2 Video).

**Table 1:** Joint excursions based on joint coordinate systems (JCS) for the shoulder, elbow, and radioulnar joint.

Joint Movement via the JCS (Axis)	Min	Max	Range	Mean	Median
Shoulder Protraction/Retraction (Z) (+/- 2°)	-97°	31°	128°	-26°	-21°
Shoulder Abduction/Adduction (Y) (+/- 1°)	-36°	3°	40°	-12°	-11°
Shoulder LAR (X) (+/- 2°)	-14°	40°	54°	17°	18°
Elbow Flexion/Extension (Z) (+/- 1°)	30°	123°	93°	81°	82°
Radioulnar LAR (X) (+/- 2°)	149°	180°	30°	174°	179°

Each JCS was based on an Euler angle ZYX rotation order that followed the right-hand rule. Numbers in parentheses indicate mean residual error in degrees for each JCS movement based on the gold standard accuracy test. At the shoulder JCS: the Z-axis measured protraction (-) and retraction (+); the Y-axis measured abduction (-) and adduction (+); and the X-axis measured medial (-) and lateral (+) long-axis rotation. At the elbow JCS, the only significant movement was at the Z-axis: flexion (-) and extension (+). At the radioulnar JCS, the only significant movement was at the long-axis rotation (LAR) at the X-axis: pronation (+) and supination (-). All joint excursions are reported relative to the reference frame.

All translations and rotations at each forelimb JCS for each trial of each rat are provided in the Supporting Information (S1 Data) as an EXCEL spreadsheet. Translations at each JCS are not reported here, although they are available in the Supporting Information. Without the scapula, the shoulder JCS translations can only indicate that the rat was moving, and not whether the humerus was translating relative to the scapula. For the elbow and radioulnar JCS, the translational movements were minimal and did not further illuminate how the radius and ulna moved relative to one another or relative to the humerus. It is conceivable that the joint center of rotation could translate as well as rotate during movement of the limb, and there is no way to precisely divide the contribution of translational and rotational movements to bone orientation. However, given that the translational movements at the elbow and radioulnar JCS are tiny (on average, submillimeter), we are confident in the overall orientation of the radius and ulna reported here.

At the beginning of the stance phase, the humerus is protracted to near vertical and the elbow is extended to approximately 70° (Fig 5). Throughout the stance phase, the humerus rotates caudally as shoulder retraction increases (Fig 5). At the forearm, the elbow first extends on initial touchdown of the manus (approximately 90°), then undergoes approximately 10° of flexion as the body shifts over the forelimb (Fig 6). Near the end of the stance phase, both shoulder retraction nears its maximal extent whereas elbow extension peaks just prior to toe-off (Figs 5 and 6). During the swing phase, the humerus briefly retracts to its maximum extent, then rapidly protracts after toe-off while the elbow maximally flexes, clearing the manus off the ground. As the swing phase ends, the humerus continues its protraction and the elbow begins extension in preparation for manus touchdown (Figs 5 and 6).

Unlike previous studies, for the first time we report three-dimensional movements of the humerus both in abduction/adduction and LAR. In fact, both shoulder abduction/adduction and LAR encompass a range of excursions approaching 40° and 55°, respectively, across our 10 trials (Table 1). During stance, the humerus is minimally abducted and in some trials adduction increased just prior to toe-off. This is perhaps not surprising given that during stance the body mass is shifted over the supporting forelimb. Towards the close of stance, just prior to swing, the humerus begins to abduct as toe-off is initiated. Throughout swing, the humerus continues to abduct, apparently to clear the body as the forelimb is rapidly protracted, with the start of adduction just prior to manus touchdown. These movements are particularly clear in the graph for Rat2 Trial 1 (S4 Fig). For LAR, the humerus is maximally rotated laterally at the beginning of stance. Throughout the stance phase, the humerus begins to rotate medially about its long-axis, a rotation that continues into approximately the first third of the swing phase. It is not until the last two-thirds of swing that humerus LAR reverses from medial to lateral. The remainder of the swing phase is lateral LAR of the humerus. These movements collectively position the forelimb closer to the rat’s center of mass during stance, and assist the forelimb in clearing the body wall during swing. The relative adduction and medial long-axis rotation at the shoulder during stance also have the combined effect of maintaining a parasagittal forearm posture with the manus placed close to the center of mass. In essence, shoulder adduction and medial long-axis rotation maintain an adducted elbow with a caudally-facing olecranon process throughout stance, which in turn maintains manus orientation.

In eutherian mammals, elbow rotation appears to be dictated by the cam-shaped trochlea of the humerus in combination with the C-shaped articular surface (semi-lunar notch) of the ulna [12,19]. These combined morphologies should restrict movements at the humero-ulnar joint (the elbow) to flexion and extension. Our data confirm this observation for rats: in all cases, the elbow rotates essentially along a single axis in parallel with the mid-sagittal plane. Mediolateral or rotational movements at the elbow either did not occur or were so miniscule (on average approximately 1°) that for all practical purposes the rat humero-ulnar articulation appears incapable of allowing any substantial movements beyond flexion and extension.

Rat long-axis rotation of the radius *in vivo* has not to our knowledge been documented previously. All three rats in all reconstructed trials show a similar pattern of radius pronation with some small individual differences (see S3 Video. Radius Pronation in Rat2). During the stance phase, the radius is rotated maximally (medially) so that it is completely crossed over the ulna from touchdown through the first half of the step cycle (Fig 4). This corresponded to approximately 174–178° of rotation from the zero position in our rat subjects. At the resolution of our data (+/- 2°), this orientation remains more or less static, although in some trials small (< 2°) medial long-axis rotational movements appear to occur. Since such small long-axis movements were difficult to detect and reconstruct, some of our trials show a static radioulnar joint value over many frames. We doubt that the radius was totally static, but at the resolution of our data, we cannot track such tiny, long-axis movements in our videos with certainty. However, it is clear that the orientation of the radius did not appreciably change during the first 50% of the step cycle (Fig 7).

At just over 50% of the step cycle, in the last 21% of stance leading to toe-off, the radius begins lateral LAR. In fact, on average the radius rotates 7–10° laterally away from its previously fully pronated orientation during this period of the step cycle. As the swing phase commences, the radius continues lateral LAR as the elbow is rapidly flexed. By approximately 75% of the step cycle (44% of swing phase), what could be called radius supination reaches its maximum extent: on average, approximately 160° compared to the zero position (Fig 7; S3 Video). Therefore, it appears that, across all three rats, the radius rotates about its long axis 10–30° from its pronated orientation during stance. We find it especially significant that the timing at which the radius rotates into “supination” closely matches elbow flexion: radius supination and elbow flexion appeared to be correlated. This is not surprising given mammalian anatomy [50]. Maximal elbow flexion is powered by the M. biceps brachii which, given the angle of its insertion into the radius, functions both as a forearm flexor and a supinator [53,54]. Just after 75% of the step cycle, the remaining swing phase sees the radius rotating medially back towards its pronated orientation as the elbow extends to prepare for touchdown and the start of a new step cycle.

## Discussion

Our data confirm and support previous reports on the basic kinematic profile of the forelimb in rats and other small therian mammals [19–22,34,52]. In general, a walking rat retains a crouched, parasagittal forelimb posture throughout the entire step cycle. We also confirmed that the humero-ulnar joint (our elbow JCS) operates essentially as a single-axis hinge joint in rats [12,19]. Our reconstructions of overground walking in *R*. *norvegicus* further substantiate the basic premise that the major forelimb movements and postures in rats are dictated proximally and refined with smaller movements distally which collectively effect manus placement and orientation [28,30].

For the first time, we demonstrated that LAR of the radius (radius pronation) occurs during a normal, overground step cycle in *Rattus norvegicus*. This finding is significant for several reasons. First, given that manus posture and radius orientation are interlinked [23,24], our data indirectly show that the full range of manus pronation and supination depend on movements of the radius in addition to those at the shoulder and elbows. Therefore, future reconstructions of forelimb movements in small therian mammals should consider the effect of radius movement on manus placement and posture. Second, our data show that elbow flexion and manus orientation are correlated in *R*. *norvegicus*: as the elbow angle becomes more acute, manus supination increases; inversely, manus pronation increases with elbow extension. Whether this pattern is particular to *R*. *norvegicus* or inherent in the forelimb mechanics of small therian mammals requires future investigations. However, given that *R*. *norvegicus* is a scansorial generalist and that the basic forelimb kinematics of small therians are conservative [21,22], we would not be surprised to find that this correlation between elbow angle and manus pronation/supination is more widespread.

Third, our data suggest that radius LAR may be related to fixation of the humero-ulnar (elbow) joint to what, for all practical purposes, amounts to a single degree of freedom (flexion/extension). Without the ability to rotate about its long-axis or abduct/adduct at the elbow, the ulna on its own can in no way contribute to manus pronation. In fact, were the radius limited so as to operate in parallel flexion and extension with the ulna, the manus would be oriented throughout the step cycle such that the digits were directed laterally. In lizards, for example, both radius and ulna parallel one another, and manus pronation is apparently accomplished, in part, through independent long-axis rotational movements of the radius and ulna about each other [55,56]. In this context, radius LAR may be essential to proper manus placement and orientation given the restricted degrees of freedom available to the ulna. In essence, manus pronation and orientation in *R*. *norvegicus* rely on a divided system of labor between the ulna and radius, both of which are proximally governed by the humerus. The radius articulates with the cranially rounded capitulum (lateral condyle) of the humerus proximally and is “parented” to the ulna. Thus, because the radius follows the flexion and extension trajectory of the ulna, it must rotate at the elbow (on the capitulum) so that during the stance phase its distal end lies medial to ulna, ensuring that the manus remains pronated while the forelimb is supporting the body.

Fourth, manus pronation is maintained in part through adduction and medial LAR of the humerus throughout the stance phase, movements which act to keep the olecranon process of the ulna caudally directed, presumably so that M. triceps brachii and other elbow extensors act continuously as anti-gravity muscles in a parasagittal plane. To our knowledge, this is the first record of humerus LAR in rats and its contribution to forelimb and manus posture. In fact, we find it significant that humerus LAR contributes to the maintenance of forelimb posture, given that this movement would not be evident because of the understandable limitations of previous studies. For example, it is often stated that small mammal kinematics remain conservative regardless of substrate. However, the standard kinematic methods by which such statements are derived are missing data on long bone LAR, which may very well change on different substrates and in different locomotor modes. As with recent studies on long bone LAR [37,38], future investigation of this motion will likely further illuminate how a crouched, parasagittal posture is maintained in small mammals across a variety of substrates, and test the hypothesis that their kinematics truly remain conservative.

Our data also have significant functional implications for fossil eutherians. The forelimbs of the two earliest known eutherian mammals, *Juramaia* and *Eomaia*, show a striking morphological resemblance to those of rats. In particular, both *Juramaia* and *Eomaia* show the presence of a well-developed, cranially rounded capitulum and a cam-shaped trochlea on the distal ends of their humeri [4,7]. Moreover, both early eutherians possess an ulna with a C-shaped articular surface (semi-lunar notch) and a radius with a rounded (but not circular) head [4,7]. These features, present in *Rattus norvegicus*, allow and constrain the range of forearm kinematics based on our reconstructions. We suggest that forelimb posture and kinematics in *Juramaia*, *Eomaia*, and other basal eutherians were grossly similar to those of rats, and that radius LAR, as well as humerus LAR, played a significant role in manus pronation and supination. If our forelimb kinematic data are applicable to basal eutherians, our results bolster the hypothesis that *Juramaia*, *Eomaia*, and their close relatives were scansorial, a locomotor habit already supported by other features of the scapula and manus [4,7].

Certainly, our conclusions about early fossil eutherian forelimb function and locomotor habits must remain tentative until future studies on *R*. *norvegicus* and other small therian forelimb kinematics can be conducted. Moreover, greater confidence in our current results require additional comparisons of three-dimensional rat forelimb kinematics through more step cycles, on a variety of substrates, and on varying perch widths using the XROMM SR technique. However, we have demonstrated the three-dimensional, interconnected modular nature of the rat forelimb in a normal, symmetrical (walking) gait. Our data in particular show that three-dimensional LAR movements within the humerus and forearms of small therians merit serious future consideration, particularly when attempting to reconstruct fossil mammal locomotion. In fact, much can also be learned from reptile and basal tetrapod locomotion [57–60] where LAR of limb elements is common. Future studies on mammalian locomotion that investigate long bone kinematics within the broader evolutionary context of non-mammal locomotor patterns promise to be fruitful.

Jenkins [20], Fischer and colleagues [21], and Fischer and Blickhan [22] have each warned about the monolithic generalization of therian mammal locomotion as “upright.” Whereas larger therians do appear to follow the “classic” inverted pendulum-like locomotor profile [61,62] typically considered mammalian, e.g. [63], smaller therians are less effected by gravity than by overcoming physical obstacles or maintaining stability on narrow perches [20–22,52]. In this context, our data, which show some significant three-dimensional rotational movements, especially at the humerus and radius, bolster the cautionary tone of previous work on small therian locomotion. We view our three-dimensional data as complementary to the legion of two-dimensional studies that have preceded ours on small therians. Ultimately, the XROMM approach [39,40] (as well as similar three-dimensional applications [26]) provides researchers with the opportunity and ability to quantify and fill significant gaps in our knowledge of mammalian forelimb functional morphology by exposing previously hidden mechanisms.

## Supporting Information

S1 Data Joint excursion data for all 10 rat trials (EXCEL). (XLS)

S1 Fig Average motion in degrees at the shoulder joint coordinate system (JCS) for a *R*. *norvegicus* step cycle showing standard deviation. Above each graph is a representation of the forelimb posture relative to the step cycle. Here, all ten trials from all three rats were binned for every 5% of the step cycle. A) All three rotational axes without standard deviation; B) Z-axis (flexion/extension); C) Green = Y-axis (abduction/adduction); D) Red = X-axis (long-axis rotation). For all graphs in this figure, Dashed lines = standard deviation.
(TIF)

S2 Fig Average motion in degrees at the elbow joint coordinate system (JCS) for a *R*. *norvegicus* step cycle showing standard deviation. Above the graph is a representation of the forelimb posture relative to the step cycle. Here, all ten trials from all three rats were binned for every 5% of the step cycle. Blue = Z-axis (flexion/extension). Dashed lines = standard deviation.
(TIF)

S3 Fig Average motion in degrees at the radioulnar joint coordinate system (JCS) for a *R*. *norvegicus* step cycle showing standard deviation. Above the graph is a representation of the forelimb posture relative to the step cycle. Here, all ten trials from all three rats were binned for every 5% of the step cycle. Radius long axis rotation (LAR, pronation) is shown in cranial view from the perspective of the ulna (the ulna appears to be stationary in these figures compared to the humerus and radius). Red = X-axis (LAR, pronation). Dashed lines = standard deviation.
(TIF)

S4 Fig Motion in degrees at the shoulder joint coordinate system (JCS) for a single rat (Rat2, Trial 1) step cycle. Above the graph is a representation of the forelimb posture relative to the step cycle. Blue = Z-axis (flexion/extension); Green = Y-axis (abduction/adduction); X-axis (long-axis rotation).
(TIF)

S5 Fig Motion in degrees at the elbow joint coordinate system (JCS) for a single rat (Rat2, Trial 1) step cycle. Above the graph is a representation of the forelimb posture relative to the step cycle. Blue = Z-axis (flexion/extension).
(TIF)

S6 Fig Motion in degrees at the radioulnar joint coordinate system (JCS) for a single rat (Rat2, Trial 1) step cycle. Above the graph is a representation of the forelimb posture relative to the step cycle. Radius long axis rotation (LAR, pronation) is shown in cranial view from the perspective of the ulna (the ulna appears to be stationary in these figures compared to the humerus and radius). Note the close synchrony between elbow flexion (Fig 6) and radius LAR. Red = X-axis (LAR, pronation).
(TIF)

S1 Table Accuracy of manual bone model registration using Scientific Rotoscoping (SR) compared with marker-based registration. (DOCX)

S2 Table Precision of manual bone model registration using repeated alignment methods. (DOCX)

S1 Video A single video of each rat trial from the perspective of both videofluoroscopes overlaid by the reconstructed animation of the polygonal bone models. (MP4)

S2 Video Rat2 Trial 1 Step cycle. (MP4)

S3 Video Radius Pronation in Rat2. Animation of radius pronation (long axis rotation) in oblique cranial view for Rat2 Trial 1.
(MP4)
