# Supplementary figures and images for: Forelimb Kinematics of Rats Using XROMM, with Implications for Small Eutherians and Their Fossil Relatives
Source: PLoS One. 2016 Mar 2;11(3):e0149377. doi: 10.1371/journal.pone.0149377 (PMC4775064; doi:10.1371/journal.pone.0149377)

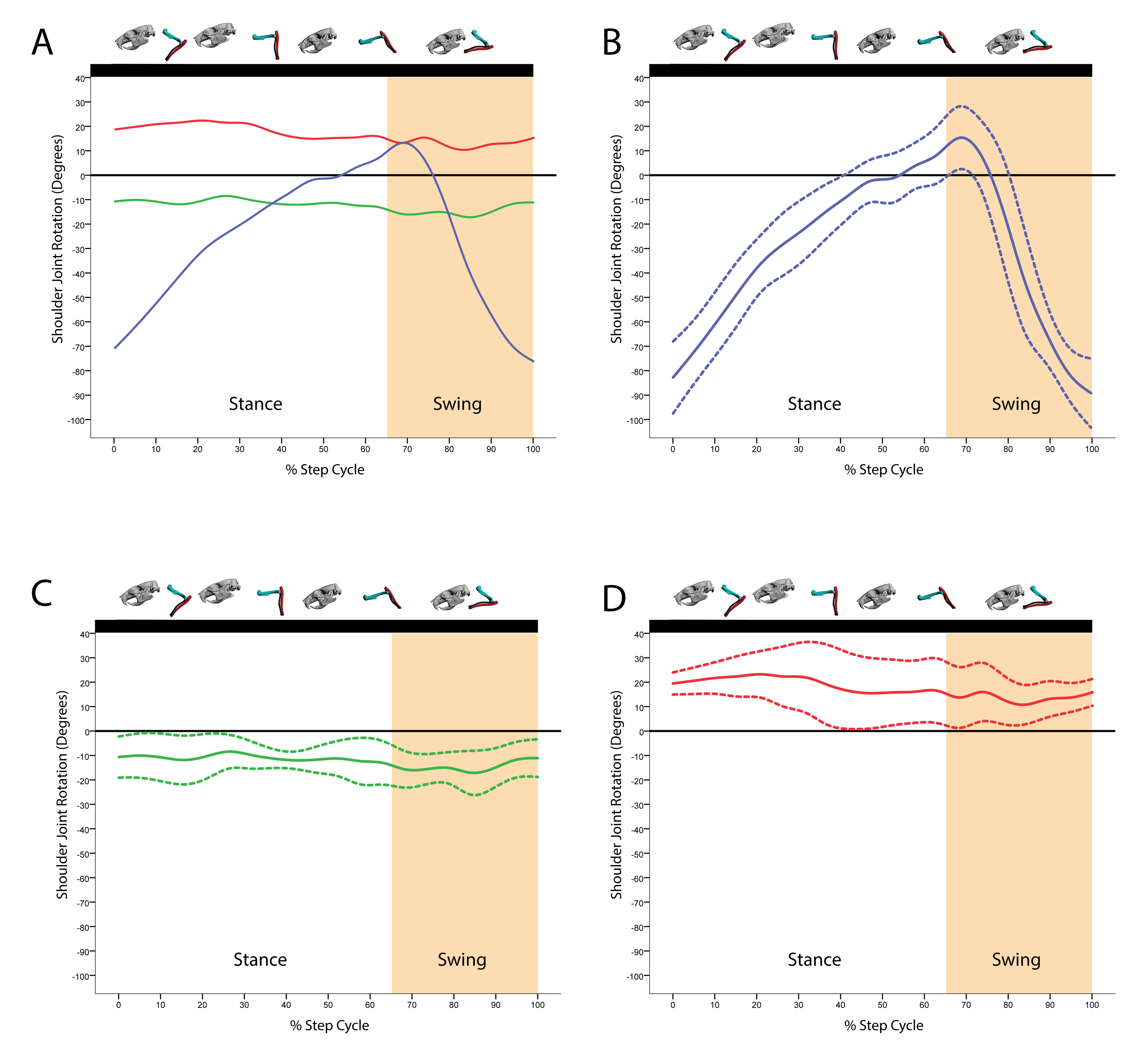

Supplement: S1 Fig — Above each graph is a representation of the forelimb posture relative to the step cycle. Here, all ten trials from all three rats were binned for every 5% of the step cycle. A) All three rotational axes without standard deviation; B) Z-axis (flexion/extension); C) Green = Y-axis (abduction/adduction); D) Red = X-axis (long-axis rotation). For all graphs in this figure, Dashed lines = standard deviation. (TIF) [file pone.0149377.s002.tif]

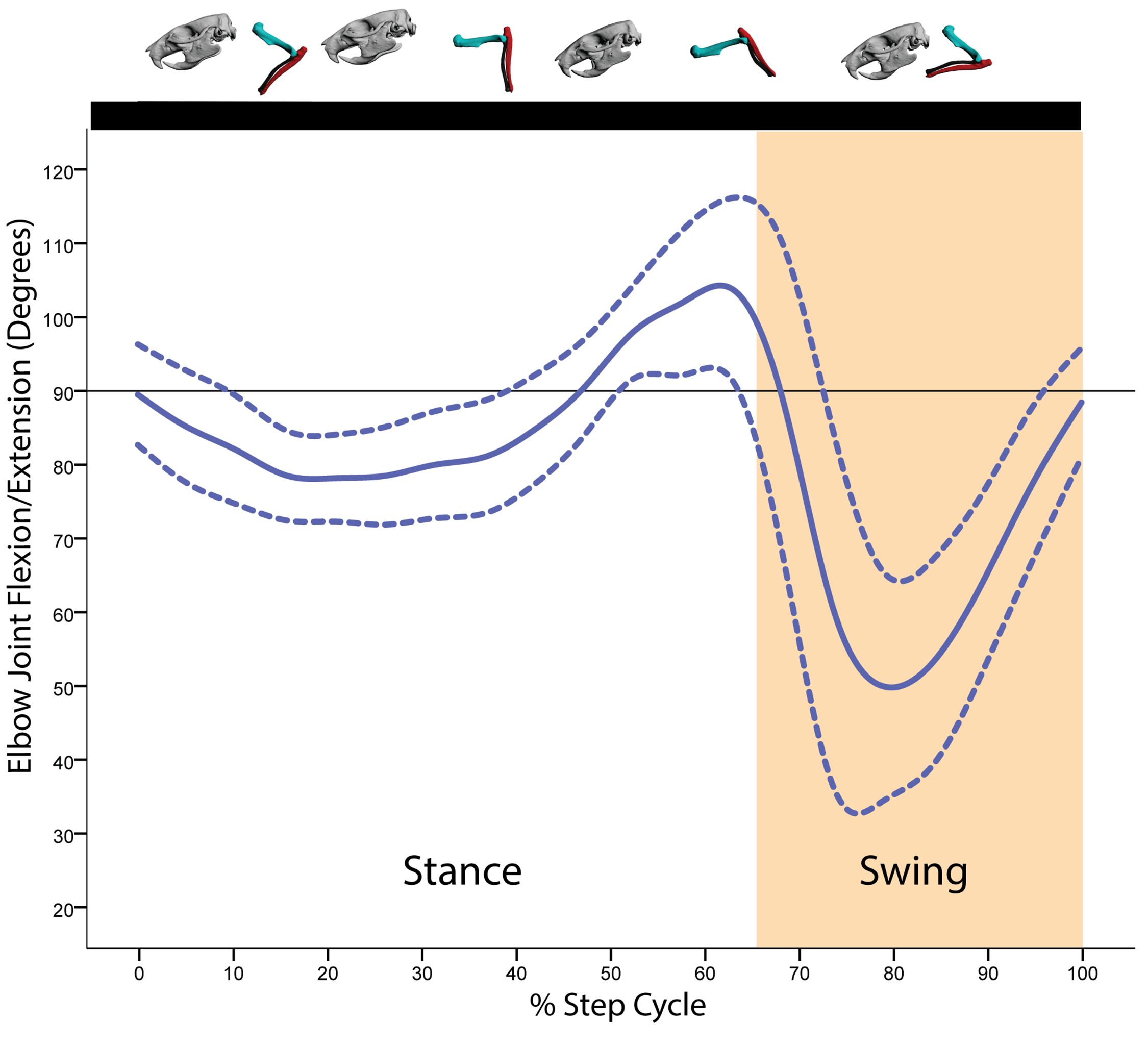

Supplement: S2 Fig — Above the graph is a representation of the forelimb posture relative to the step cycle. Here, all ten trials from all three rats were binned for every 5% of the step cycle. Blue = Z-axis (flexion/extension). Dashed lines = standard deviation. (TIF) [file pone.0149377.s003.tif]

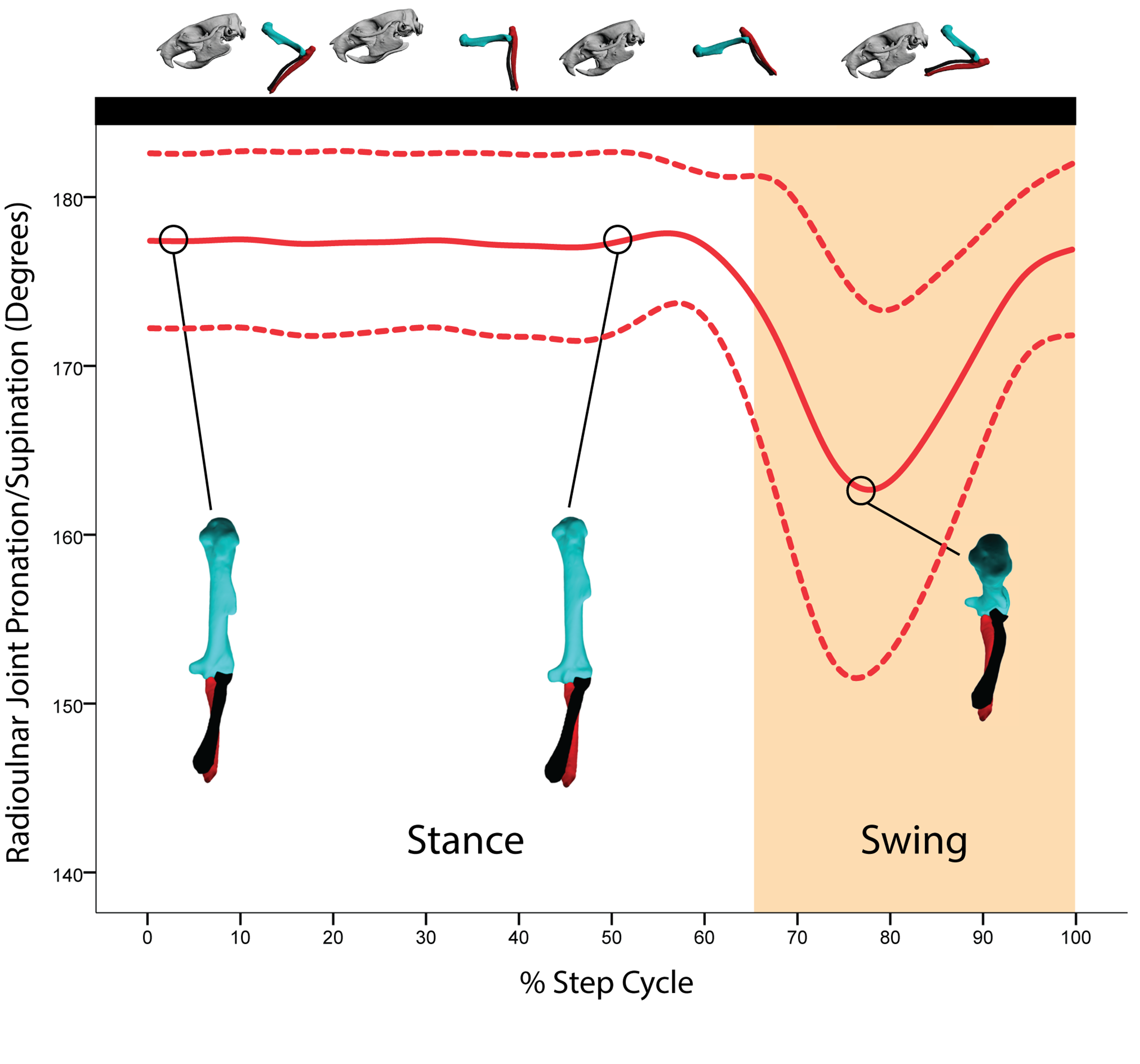

Supplement: S3 Fig — Above the graph is a representation of the forelimb posture relative to the step cycle. Here, all ten trials from all three rats were binned for every 5% of the step cycle. Radius long axis rotation (LAR, pronation) is shown in cranial view from the perspective of the ulna (the ulna appears to be stationary in these figures compared to the humerus and radius). Red = X-axis (LAR, pronation). Dashed lines = standard deviation. (TIF) [file pone.0149377.s004.tif]

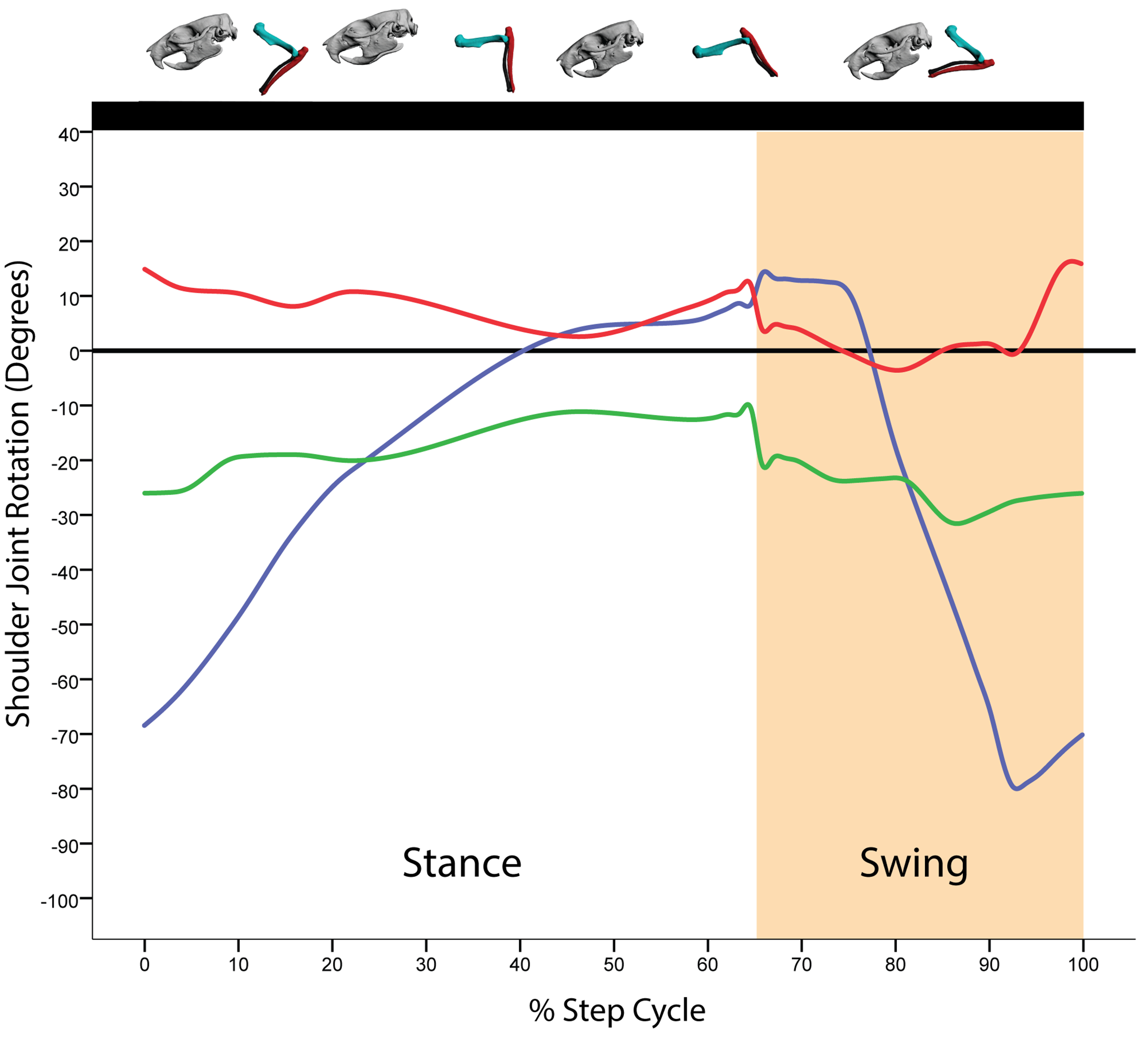

Supplement: S4 Fig — Above the graph is a representation of the forelimb posture relative to the step cycle. Blue = Z-axis (flexion/extension); Green = Y-axis (abduction/adduction); X-axis (long-axis rotation). (TIF) [file pone.0149377.s005.tif]

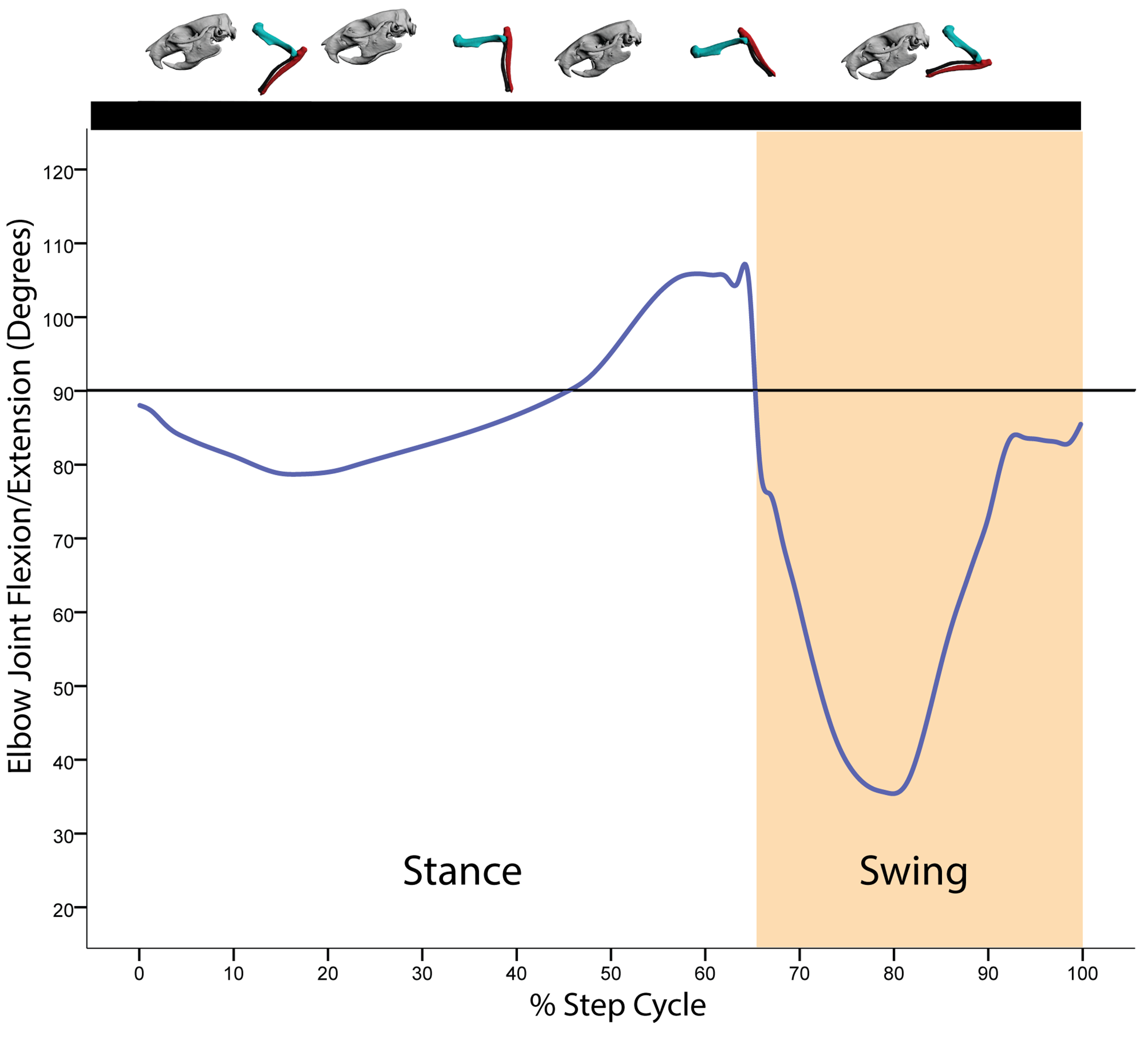

Supplement: S5 Fig — Above the graph is a representation of the forelimb posture relative to the step cycle. Blue = Z-axis (flexion/extension). (TIF) [file pone.0149377.s006.tif]

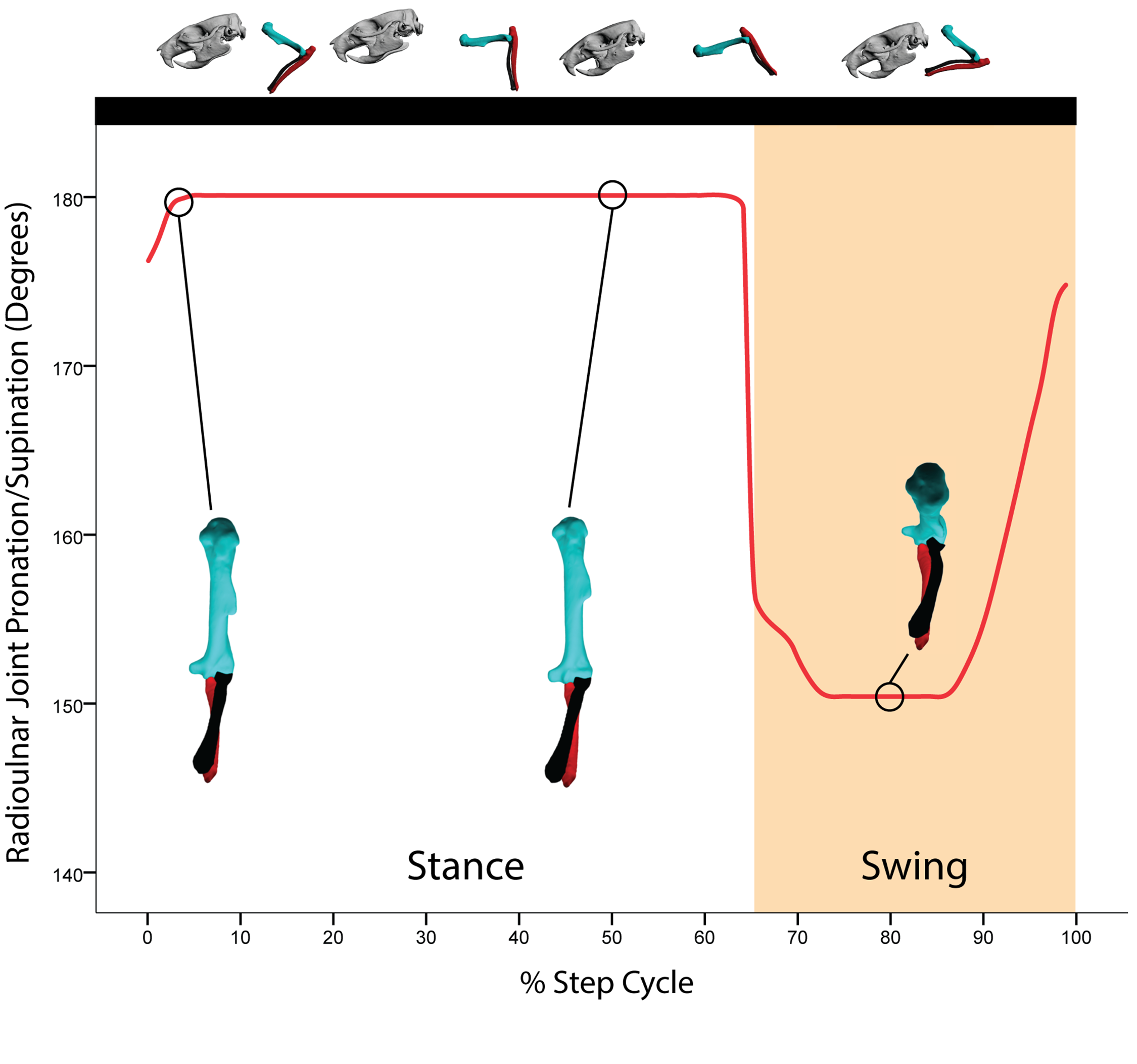

Supplement: S6 Fig — Above the graph is a representation of the forelimb posture relative to the step cycle. Radius long axis rotation (LAR, pronation) is shown in cranial view from the perspective of the ulna (the ulna appears to be stationary in these figures compared to the humerus and radius). Note the close synchrony between elbow flexion (Fig 6) and radius LAR. Red = X-axis (LAR, pronation). (TIF) [file pone.0149377.s007.tif]
