# Supplementary material for: Forelimb Kinematics of Rats Using XROMM, with Implications for Small Eutherians and Their Fossil Relatives
Source: PLoS One. 2016 Mar 2;11(3):e0149377. doi: 10.1371/journal.pone.0149377 (PMC4775064; doi:10.1371/journal.pone.0149377)
Supplement: S1 Table — (DOCX) [file pone.0149377.s008.docx]

S1 Table. Accuracy of manual bone model registration using Scientific Rotoscoping (SR) compared with marker-driven registration (translations in cm and rotations in degrees). To determine the accuracy of manual registration used in this study (SR), the skull, sternum, humerus, ulna, and radius of a cadaver rat were implanted with tantalum beads that could be automatically tracked using the XMA Lab software. The forelimb of the cadaver was puppeted and the coordinates of the selected elements were tracked. The resulting known orientations (the marker driven gold standard) were then compared with the same scene in which the elements were manually registered using SR. Accuracy is reported as the mean residuals at the joints (standard deviation in parentheses) comparing the marker-based measurements with manual registration. Abbreviations: ZYX, translational movements in cm and rotational movement in degrees at each joint.

| Joint | Movement | Z | Y | X |
| --- | --- | --- | --- | --- |
| Skull / Sternum* | Translation  Rotation | 0.13 (0.07)  0.40 (0.25) | 0.07 (0.05)  1.12 (0.84) | 0.06 (0.05)  1.77 (1.50) |
| Shoulder | Translation  Rotation | 0.09 (0.07)  2.26 (1.22) | 0.08 (0.06)  1.76 (1.24) | 0.08 (0.06)  2.34 (1.48) |
| Elbow | Translation  Rotation | 0.03 (0.02)  1.39 (1.35) | 0.04 (0.02)  3.18 (2.46) | 0.03 (0.02)  1.95 (2.14) |
| Radioulnar Joint | Translation  Rotation | 0.05 (0.04)  3.56 (1.52) | 0.02 (0.02)  2.34 (1.66) | 0.09 (0.06)  1.82 (1.67) |

*This joint measured movements of the sternum, a proxy for the body midline, relative to the skull.
