# Supplementary material for: Forelimb Kinematics of Rats Using XROMM, with Implications for Small Eutherians and Their Fossil Relatives
Source: PLoS One. 2016 Mar 2;11(3):e0149377. doi: 10.1371/journal.pone.0149377 (PMC4775064; doi:10.1371/journal.pone.0149377)
Supplement: S2 Table — (DOCX) [file pone.0149377.s009.docx]

S3. Table. Precision of manual bone model registration using repeated alignment methods. For each rat, a frame was selected from one of the trials and the model was re-registered (aligned) to that frame 10 times. We tested the most proximal (shoulder) and most distal (radioulnar) joints in our rig. Precision of the shoulder is crucial because both its translations and rotations substantially affect the orientation of the radius and ulna elements distal to the humerus. Precision of the radiounlar joint was also critical to demonstrate whether significant long-axis rotation (LAR) of the radius occurs relative to ulna. The values listed are the grand means of all trials for all rats. Since the radius is parented to the ulna, its translations follow the elbow and shoulder. Therefore, only the rotation values are reported for the radiounlar joint. Abbreviations: Trans, translational movements in cm; Rot, rotational movements in degrees; CI, confidence interval.

| Joint | Test | TransX | TransY | TransZ | RotX | RotY | RotZ |
| --- | --- | --- | --- | --- | --- | --- | --- |
| Shoulder | SD | 0.05 cm | 0.07 cm | 0.03 cm | 1.51° | 1.17° | 1.99° |
|  | 95% CI | 0.03 cm | 0.04 cm | 0.02 cm | 1.23° | 0.96° | 1.62° |
| Radioulnar | SD |  |  |  | 0.37° | 0.36° | 0.91° |
|  | 95% CI |  |  |  | 0.23° | 0.22° | 0.56° |
